# Promoting racial equity in digital health: applying a cross-disciplinary equity framework

**DOI:** 10.1038/s41746-023-00747-5

**Published:** 2023-01-11

**Authors:** Marium M. Raza, Kaushik P. Venkatesh, Joseph C. Kvedar

**Affiliations:** Harvard Medical School, Boston, MA USA

**Keywords:** Health policy, Ethics

## Abstract

Even as innovation occurs within digital medicine, challenges around equity and racial health disparities remain. Golden et al. evaluate structural racism in their recent paper focused on reproductive health. They recommend a framework to Remove, Repair, Restructure, and Remediate. We propose applying the framework to three areas within digital medicine: artificial intelligence (AI) applications, wearable devices, and telehealth. With this approach, we can continue to work towards an equitable future for digital medicine.

Equity has become a prime concern in digital health’s promise to make medical care more efficient and effective^1^. Historically, racism has been a key barrier to equity in health care. Specifically, structural and systemic discrimination have resulted in disparities in health before patients even reach medical care, and then disparities exist in the care provided after patients actually get into the clinic^2^. In their 2005 report, the National Academy of Medicine concluded “some people in the United States were more likely to die from cancer, heart disease, and diabetes simply because of their race or ethnicity, not just because they lack access to health care”^3^. These racial disparities remain entrenched within our healthcare system. Even today, Black women of childbearing age die from complications of pregnancy at nearly four times the rate of White women^4^.

Golden et al. posit that while medical technology continues to advance, actions at multiple levels are required to dismantle structural racism and address healthcare disparities^5^. In examining the impact of racism specifically on reproductive health, they recommend using the Remove, Repair, Restructure, Remediate (R4P) approach supported by McLemore’s Retrofit, Reform, and Reimagine (3 R) model^6^. The R4P approach involves *removing* power structures that promote inequality, *repairing* systems given the historical context of racism, *restructuring* policies and institutions, and *remediating* immediate needs.

A similar approach should be applied more broadly to center equity in the development and implementation of digital health innovations. We argue that the R4P/3 R framework should be applied to three key areas transforming digital medicine: artificial intelligence (AI) applications, wearable devices, and telehealth.

## AI algorithms

AI algorithms are being applied to health care in various fields including clinical research, decision support, and targeted therapy^7^. There is concern that the way these algorithms are developed may reproduce racial biases on a variety of levels. AI algorithms are created by people within the confines of structural inequities, so bias can be enmeshed in the decisions made by developers, e.g. the outcome variables tracked. Additionally, the datasets used to train AI algorithms may be unrepresentative of marginalized groups, who are already underrepresented in clinical research datasets^8^.

One 2019 study found evidence of racial bias in a widely used commercial algorithm that tracked health risk. In the algorithm, sicker Black patients were assigned the same level of risk as healthier White patients^9^. Upon evaluation, the driver of bias was that the algorithm used health costs as a proxy for health needs, and less money is spent on Black patients who have the same level of need.

Applying the R4P/3 R approach, fixing such algorithmic bias is an example of *remediating* immediate needs by retrofitting currently used technologies with racial equity goals in mind. More broadly, many algorithms deployed on large scales are proprietary, making them difficult to evaluate^10^. *Removing* power structures would require increased transparency so algorithms can be critically evaluated for racial equity. This evaluation should include an algorithm’s training data, objective function, and prediction methodology. *Restructuring* policies and institutions would involve creating the infrastructure to evaluate algorithms from specific equity criteria. A recent study found that an AI tool trained on medical imaging could unexpectedly predict race^11^. Models that result in such flawed associations should be screened for in the approval process and reworked.

## Consumer wearables

Consumer wearable devices, designed to promote healthy living and alert consumers based on real-time data, may reproduce racial disparities as well. Wearable devices are created within structural confines, so the technology itself can be biased^12^. For example, many consumer devices use photoplethysmographic (PPG) green light signaling to estimate changes in heart rate, rhythm, and even sleep architecture. There is evidence that PPG measurements may be less accurate for darker skin tones, depending on measurement condition and test data sets^13–15^. In addition, there are significant racial disparities in terms of access to wearable devices. A study from 2022 showed that wearable devices, along with other digital technologies, are not as widely used in low-income and minority populations^16^. The study highlights cost and education as significant factors affecting access and use of wearables.

In this case, *removing* power structures would focus on increasing access to and education about wearable devices in marginalized communities. *Restructuring* would involve evaluating racial equity within the FDA approval process. Currently, the FDA 510(k) clearance process, which is the way to FDA approval for most wearable devices, only requires equivalent safety and efficacy to products that are already available^17^. As initial wearable device studies did not focus on equity and technology validity across racial phenotypes, deficits in the representation of marginalized populations have continued. Restructuring should also include transparency about which devices may be less accurate; for example, some manufacturers already recommend only using their device in light skin tones or at rest^18^. *Repairing* and *remediating* would involve increased research funding to evaluate and create devices that accurately function across different racial and ethnic phenotypes to repair existent disparities.

## Telehealth

Finally, while the growing field of telehealth has shown promise for increasing access to healthcare, racial disparities persist. One multi-clinic study found utilization gaps in telemedicine uptake among racial minorities during the COVID-19 pandemic^19^. The study notes that reduced broadband access, disparities in health literacy, and patient preference may be factors affecting telemedicine uptake among minority populations. Indeed only 66% of African American and 61% of Hispanic households have access to broadband compared to 79% of White households in the US^20^. These findings suggest that in its current form, telehealth services have the unfortunate potential to exacerbate existing disparities. Another study showed that racial minority patients receiving services in urban areas were the most vulnerable to being lost to follow-up in the transition to telemedicine services, and posited an association between geographic location and insurance access^21^.

To *remove* power structures, researchers and providers should develop interventions to remediate racial disparities in telehealth usage. For example, providers can enlist the use of remote interpreters and health systems can provide technology education for patients with limited health literacy. One promising framework to remove power structures, and work towards removing health disparities using telehealth has already been proposed by the American Telemedicine Association^22^. *Restructuring* must involve an evaluation of the financial incentives for insurance plans to cover telemedicine visits, establish racial equity as a key quality metric for telemedicine, and create programs to ensure broadband access for low-income users. Providers can also help extend public services like the Affordable Connectivity Program that provides $30/month for lower-income households to access broadband^23^. In this case, as the shift to telehealth services has already skyrocketed in the aftermath of the COVID-19 pandemic, *remediating* the aggravation of existing disparities is key. Institutions should actively assess whether their most vulnerable patients have access to and education about telehealth services.

Ultimately digital health technology, whether it is AI algorithms, wearable devices, or telemedicine, does not inherently contain bias. Indeed, technology has the potential to reduce racial disparities when used appropriately to increase access to safe, effective health care. Without a focus on equity, however, racial disparities will only continue to be perpetuated within innovation. The R4P approach provides an important framework that should be applied systematically to applications of digital health technology, to help create sustainable change towards an equitable future for digital medicine.

See Table 1.

**Table 1:** R4P Model for Digital Medicine Innovation.

	AI Algorithms	Wearables	Telehealth
R4P Model			
Remove	- correct bias in algorithms - recall applications on market dangerous to health equity	- increase health literacy/education about wearables	- increase structural access to telehealth-enabling technology, e.g. broadband, video/audio device
Repair	- assess barriers to representation in model training data from diverse populations - train developers on sources, dangers, and prevention of biased algorithms	- assess barriers to access for wearables - increase transparency about wearable device accuracy/efficacy	- telehealth training and literacy initiatives - build cultural competence + technical skills in providers who use telehealth with diverse patients
Restructure	- establish equity/diversity criteria for datasets and approval - establish organizational incentives, e.g. provider-developer agreements	- health equity criteria for FDA approval process - public funding or payer coverage for access to wearables	- coverage of telehealth services - racial equity as quality metric for telehealth
Remediate	- establish liability for algorithmic bias and racism - establish protection of marginalized groups - consider new ways to recruit diverse populations	- increase research funding for inclusive wearable devices - consider new ways to recruit diverse populations	- actively assess current/ongoing adoption of telehealth from health equity standpoint

## Data Availability

No datasets were produced or analyzed for this article.

## Code availability

No computer code was produced or analyzed for this article.
